# Maxillofacial and neck trauma: a damage control approach

**DOI:** 10.1186/s13017-015-0022-9

**Published:** 2015-07-07

**Authors:** Amir A. Krausz, Michael M. Krausz, Edoardo Picetti

**Affiliations:** Department of Oral & Maxillofacial Surgery, Rambam Health Care Campus, Haifa, Israel; Department of Surgery, Hillel Yaffe Medical Center, Hadera, Technion-Israel Institute of Technology, Haifa, Israel; Division of Anesthesia and Intensive Care, Azienda Ospedaliero-Universitaria di Parma, Parma, Italy

**Keywords:** Maxillofacial trauma, Neck trauma, Damage control surgery, Damage control resuscitation, Massive transfusion

## Abstract

Severe maxillofacial and neck trauma exposes patients to life threatening complications such as airway compromise and hemorrhagic shock. These conditions require rapid actions (diagnosis and management) and a strong interplay between surgeons and anesthesiologists. Effective airway management often makes the difference between life and death in severe maxillofacial and neck trauma and takes initial precedence over all other clinical considerations. Damage control strategies focus on physiological and biochemical stabilization prior to the comprehensive anatomical and functional repair of all injuries. Damage control surgery (DCS) can be defined as the rapid initial control of hemorrhage and contamination, temporary wound closure, resuscitation to normal physiology in the intensive care unit (ICU) and subsequent reexploration and definitive repair following restoration of normal physiology. Damage control resuscitation (DCR) consists mainly of hypotensive (permissive hypotension) and hemostatic (minimal use of crystalloid fluids and utilization of blood and blood products) resuscitation. Both strategies should be administered simultaneously in all of these patients.

## Review

### Introduction

**Fig. 1:**
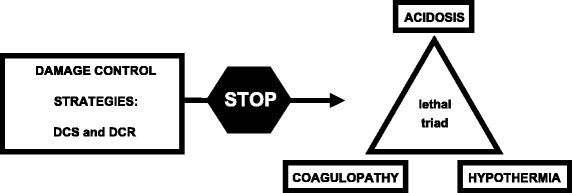
Role of damage control strategies (DCS and DCR) in severely injured patients. DCS = damage control surgery. DCR = damage control resuscitation

Severe maxillofacial and neck trauma exposes patients to life threatening complications such as airway compromise and hemorrhagic shock [1, 2]. These conditions require not only a rapid recognition and management but also a strong interplay between surgeons, anesthesiologists and other relevant medical personnel. Hemorrhage represents one of the leading causes of death following trauma [3, 4]. After the initial insult, the combined occurrence of coagulopathy, hypothermia, and acidosis (“the lethal triad”) further contributes to the poor prognosis of severely exsanguinating trauma patients [5]. Over the last years, new strategies termed DCS and DCR have gained popularity in the management of injured patients [5–7]. Damage control strategies focus on physiological and biochemical stabilization prior to the comprehensive anatomical and functional repair of all injuries [5–7]. DCS can be defined as the rapid initial control of hemorrhage and contamination, temporary wound closure, resuscitation to normal physiology in the ICU and subsequent re-exploration and definitive repair following restoration of normal physiology [5, 8]. DCR, defined as nonsurgical strategies utilized to prevent or reverse the effects and outcomes of “lethal triad”, consists mainly of hypotensive (permissive hypotension) and hemostatic (minimal use of crystalloid fluids and utilization of blood and blood products) resuscitation [5–7]. DCR and DCS should be administered simultaneously with close collaboration between all medical personnel involved in the patient’s treatment (Fig. 1). Damage control strategies have been applied initially to abdominal trauma [9] and subsequently to other fields such as thoracic surgery [10] and orthopedics [11]; relatively high survival rates with these strategies have been reported [5–7, 12]. Case reports demonstrate the benefits of applying damage control principles to maxillofacial and neck trauma patients [13, 14]. Appropriate selection of patients that can benefit from DCS is very important. In severe trauma patients with serious physiological derangement, attempts of primary definitive surgical management can inevitably lead to poor outcome. In contrast, inappropriate use of DCS may expose patients to additional unnecessary procedures which associated costs and complications [6, 15–17]. In addition to injury patterns, indications for the application of a damage control strategy are mainly related to: 1) significant bleeding requiring massive transfusion, 2) severe metabolic acidosis (pH < 7.30), 3) hypothermia (T < 35.8 °C), 4) estimated operating time > 90 min, 5) coagulopathy and 6) lactate > 5 mmol/l [6, 18–20].

### Damage control resuscitation

#### Massive transfusion

Massive transfusion (MT) may be defined as the rapid transfusion of a large volume of blood products to a patient with severe hemorrhage [21]. Classically MT is defined as the transfusion of ≥ 10 red blood cell (RBC) units within 24 h [21]. Other definitions are in use: transfusion of > 4 RBC units over 1 h with anticipation of continued need and replacement of > 50 % of the total blood volume (normal adult male near 70 ml/kg, female near 60 ml/kg) with blood products within 3 h [21]. The creation of an institutional protocol for MT transfusion could prove very useful in facilitating communication between different hospital services and avoiding delays in care of exsanguinating trauma patients. Early recognition of patients that may benefit from a MT is a fundamental step in improving outcome; however, in certain situations it is unclear which bleeding patient will require MT [21]. In this regard, several scores such as Trauma Associated Severe Hemorrhage (TASH) score [22] and ABC score [23] have been validated and utilized (Table 1).

**Table 1:** Scores for massive transfusion

TASH score	1. SBP: < 100 mmHg = 4 pts, < 120 mmHg = 1 pt
(0–28 pts, Increasing TASH-scores were associated with an increasing probability for MT requirement)	2. Hb: < 7 g/dl = 8 pts, < 9 g/dl = 6 pts, < 10 g/dl = 4 pts, < 11 g/dl = 3 pts, < 12 g/dl = 2 pts
	3. intra-abdominal fluid : 3 pts
	4. complex long bone and/or pelvic fractures: AIS 3 or 4 = 3 pts, AIS 5 = 6 pts
	5. HR: > 120 bpm = 2 pts
	6. BE: < -10 mmol/l = 4 pts, < -6 mmol/l = 3 pts, < -2 mmol/l = 1 pt
	7. gender: male = 1 pt
ABC score	1. penetrating mechanism: no = 0 pt, yes = 1 pt
(0–4 pts, a score of 2 or greater was used to predict the need for MT)	2. SBP < 90 mmHg: no = 0 pt, yes = 1 pt
	3. HR > 120 bpm: no = 0 pt, yes = 1 pt
	4. positive FAST: no = 0 pt, yes = 1 pt

*TASH* trauma associated severe hemorrhage, *SBP* systolic blood pressure, *pts* points, *pt* point, *Hb* hemoglobin, *AIS* abbreviated injury score, *HR* heart rate, *bpm* beats per minute, *BE* base excess, *FAST* focused assessment with sonography for trauma, *MT* massive transfusion

#### Hypotensive resuscitation

Early and aggressive fluid administration to restore blood volume may be associated with several adverse effects such as: 1) dislodgement of blood clots (by increasing hydrostatic pressure on the wound), 2) dilution of coagulation factors and 3) hypothermia [5–7]. Permissive hypotension represents a strategy through which blood pressure is maintained at low values (ex. by restricting/avoiding fluid supplement) until hemorrhage is controlled, while accepting a limited period of suboptimal perfusion of end organs [5–7].

Recent guidelines [24] suggest the maintenance of different SBP values (for TBI and non-TBI patients) until major hemorrhage control has been achieved during the initial phase following trauma (Table 2).

**Table 2:** Hemostatic/Hemodynamic resuscitation following major trauma

Tranexamic Acid	within 3 h of injury loading dose 1 g over 10 min, followed by 1 g over 8 h
Ionized Calcium Levels	maintain in normal ranges during MT
Plasma : RBC	at least 1:2 (preferably 1:1) maintain Hb levels: 7–9 g/dl maintain coagulation parameters (repeated monitoring of PT, aPTT, fibrinogen levels, platelets count, viscoelastic testing) in normal ranges during MT
Fibrinogen	3–4 g administer in case of thromboelastometric signs of a functional fibrinogen deficit or a plasma fibrinogen level of less than 1.5 to 2 g/l
Platelet Count	50 × 10^9^/l if ongoing bleeding and/or TBI: 100 × 10^9^/l initial dose 4–8 single platelet units or 1 aphaeresis pack
Blood Pressure	SBP: 80 to 90 mmHg until hemorrhage control (no TBI) if severe TBI (GCS ≤ 8) MAP ≥ 80 mmHg
	consider rFVIIa if major bleeding and traumatic coagulopathy persist despite maximal attempts to stop bleeding
	in case of pre-trauma therapeutic anticoagulation or antiplatelets drugs consider specific treatment (ex. desmopressin, PCC, etc)

*MT* massive transfusion, *Hb* hemoglobin, *PT* prothrombin time, *aPTT* activated partial thromboplastin time, *TBI* traumatic brain injury, *SBP* systolic blood pressure, *MAP* mean arterial pressure, *GCS* Glasgow Coma Scale, *rFVIIa* recombinant activated coagulation factor VII, *PCC* prothrombin complex concentrate

#### Hemostatic resuscitation

Generally, post-traumatic coagulopathy was considered to be the result of 1) loss of procoagulant factors (caused by consumption and bleeding), 2) hemodilution (caused by fluid resuscitation) and 3) coagulatory dysfunction (due to acidosis and hypothermia) [5]. However, an early form of coagulopathy was found mainly in patients with hypoperfusion [7, 25, 26]. This “acute traumatic coagulopathy” is characterized by an up-regulation of endothelial thrombomodulin, which forming complexes with thrombin, promotes anticoagulation [26] as well as by endothelial dysfunction-induced hyperfibrinolysis [25]. This form of coagulopathy is present in the most severely injured patients and is usually associated with poor outcomes [24, 27, 28]. Based on the above, hemostatic resuscitation could be considered as the utilization of blood and blood products in the early treatment phases of traumatic coagulopathy. Adequate detection and treatment of post-traumatic coagulopathy mandates early and repeated monitoring of prothrombin time (PT), activated partial thromboplastin time (aPTT), fibrinogen levels and platelets count [24]. Moreover, the utilization of recently introduced viscoelastic testing, such as thrombelastography, may be useful for better characterization of an existing coagulopathy and management of haemostatic therapy [21, 24]. An ideal test (rapid, highly sensitive, etc.) does not yet exist. For this reason, in the very early acute phases of trauma management, the decision to initiate clotting factor replacement is based mainly on clinical considerations [5]. In regard to hemostatic resuscitation following major trauma, the reciommedations of recently updated European guidelines [24] are summarized in Table 2.

Recently, the administration of RBC:plasma:platelets at 1:1:1 ratio (more closely resembling whole blood) during MT has been supported by several military and civilian studies [21, 29–32]. The Prospective Observational Multicenter Major Trauma Transfusion (PROMMT) Study [31], involving 1245 trauma patients (905 in the cohort analysis group), sought to examine the relationship between in-hospital mortality to early transfusion of plasma and/or platelets and to time-varying plasma: RBC and platelet: RBC ratios. The authors hypothesized that early transfusion of plasma and platelets in higher ratios would be associated with decreased in-hospital mortality in bleeding patients. This study shows that an early infusion of higher plasma and platelet ratios was associated with decrease mortality within 6 h of admission. In the first 6 h, patients with ratios lower than 1:2 were 3 to 4 times more likely to die than patients with ratios of 1:1 or higher. A 1:1 ratio of plasma and platelets was associated with decreased early mortality compared with lower ratios. The Pragmatic Randomized Optimal Platelet and Plasma Ratios (PROPPR) trial [32] is a prospective randomized pragmatic study which compares a 1:1:1 ratio of plasma: platelets: RBCs with a 1:1:2 ratio in 680 severely injured patients predicted to receive MT at 12 trauma centers in North America. Early administration of plasma, platelets, and red blood cells in a 1:1:1 ratio compared with a 1:1:2 ratio did not result in significant differences in mortality at 24 h or at 30 days. However, more patients in the 1:1:1 group achieved hemostasis and fewer experienced death due to exsanguination at 24 h.

### Damage control surgery

Severe maxillofacial and neck trauma, both blunt and penetrating, may be sustained in various military and civilian circumstances. Civilian trauma settings such as motor vehicle accidents (MVAs), falls, altercations, sports and occupational injuries, etc. may cause blunt trauma of various degrees, usually resulting in closed fractures and soft tissue lacerations or mild to moderate penetrating trauma. Blast injuries (tertiary effect) may cause variable degrees of maxillofacial and neck blunt trauma in military combat or civilian terrorist conflicts. In such circumstances, severe penetrating maxillofacial and neck injuries are often encountered, usually inflicting complex lacerations, open fractures and wounds complicated by tissue avulsions and wounds [33]. Nowadays, modern body armor and improved cranial protection allow a relatively high percentage of military head and neck trauma casualties to survive their initial injuries pending arrival at a Level III trauma care center. Civilian MVA casualties also portray high survival rates owing to modern car safety measures such as airbags, early warning systems, etc. Civilian head and neck casualties nowadays benefit from high relative proximity of most civilian injuries to Level III trauma care centers when compared to previous eras, thus enhancing emergency room (ER) arrival survival rates. Initial management of these injuries involves: 1) airway management 2) control of hemorrhage 3) prevention of disability from central nervous system injuries. Penetrating maxillofacial and neck injuries result in a complex of lacerations, open fractures, profuse bleeding, tissue avulsions, eye injuries and burns. These highly visible injuries, although bloody, are rarely the sole cause of hemorrhagic shock. The critical immediate life-threat following maxillofacial injury results from airway compromise due to oropharyngeal bleeding, swelling, and loss of mandibular structural integrity. The airway is maintained and secured by oro-tracheal intubation, cricothyroidotomy, or surgical tracheotomy. Once the airway is secured, direct pressure and aggressive packing of open bleeding wounds will control all but the most catastrophic hemorrhages. Previously stated rationale dictates that direct manual pressure and aggressive packing of open bleeding wounds or bleeding nasal and oropharyngeal cavities should be implemented without further delay in order to minimize further blood loss. External fixation of unstable anterior mandibular fractures using available means (orthopedic pin fixators, wire ligatures etc.) may assist in preventing airway compromise, as well as reduce profuse bleeding, pain and morbidity often associated with such injuries. More significant bleeding can be controlled by angiographic embolization or selective ligation of the external carotid artery, while simultaneously protecting the brain, cervical spine, and the eyes from further injury. Early fixation of a flail mandible is also mandatory because it may destabilize tongue musculature insertions thus compromising the airway and also cause bleeding, significant pain and morbidity. Head and neck injuries should be copiously irrigated, wound contaminants removed, clearly nonviable tissue fragments should be debrided and the wounds covered to prevent further contamination. Suturing of soft tissue lacerations covering underlying bone fractures should be deferred so that such fractures should not be overlooked during subsequent examination of the patient.

#### Maxillofacial anatomy

Maxillofacial anatomy consists of the bony and soft tissue structures anterior and inferior to the base of the skull, from the ears forward and from the eyebrows down to the chin. The tongue is attached to the forward projecting mandible, which supports the patency of the airway. Beneath the skin, critical structures at risk from penetrating trauma are the major salivary glands, salivary ducts, and major facial nerve branches, and blood vessels. Unrecognized injuries to these structures may result in significant morbidity [34]. The midface extends between the eyebrows and base of nose, which contains the air-filled paranasal sinuses. The bones encasing the sinus cavities are thin and lined by mucosa. Projectiles can easily perforate and transverse the midface leading to subsequent infections and profuse bleeding from terminal branches of the maxillary artery. Ocular injuries, vascular injuries, and intracranial penetration are critical associated injuries following penetrating trauma to the midface. The lower face is the area lies from the base of the nose to the chin including the tooth-bearing parts of the maxilla and the entire mandible. The lower face skeleton is composed of thick cortical bone and dense dental structures. The tongue, confined by these hard structures, is at risk for severe injury, bleeding and swelling leading to airway obstruction.

#### Neck anatomy

It is useful to divide the anatomical structures of the neck into five major functional groups, in order to facilitate and ensure a comprehensive assessment and surgical approach:

1. Airway - pharynx, larynx, trachea, lung.
2. Major blood vessels - carotid artery, innominate artery, aortic arch, jugular vein, subclavian vein
3. Gastrointestinal tract - pharynx, esophagus.
4. Nerves - spinal cord, brachial plexus, cranial nerves, peripheral nerves.
5. Bones - mandibular angles, styloid processes, cervical spine.

The platysma defines the border between the superficial and the deep structures of the neck. If a wound does not penetrate deep to the platysma, it is not classified as a significant penetrating neck wound. As transverse cervical veins running superficial to the platysma may bleed profusely when severed, they are easily controlled by direct pressure, and can be managed by a simple ligature. The sternocleidomastoid muscle divides the neck into the posterior triangle which contains the spine and muscles, and the anterior triangle which contains the vasculature, nerves, airway, esophagus and salivary glands.

When evaluating penetrating neck injuries, the neck is divided into three anatomic zones for purposes of initial assessment and management planning:

- Zone I: extends between the clavicle/suprasternal notch and the cricoid cartilage (including the thoracic inlet). Surgical access to this zone may require thoracotomy or sternotomy. Major arteries and veins, trachea and nerves, esophagus, lower thyroid and parathyroid glands and thymus are located in this zone.
- Zone II: lies between horizontal lines drawn at the level of the cricoid cartilage and the angle of the mandible. It contains the internal and external carotid arteries, jugular veins, pharynx, larynx, esophagus, recurrent laryngeal nerves, spinal cord, trachea, upper thyroid and parathyroid glands.
- Zone III: extends between the angle of the mandible and base of skull. It contains the extracranial carotid and vertebral arteries, jugular veins, cranial nerves IX–XII and sympathetic nerve trunk.

#### Airway control and breathing support in maxillofacial trauma

Effective airway management often makes the difference between life and death in severe maxillofacial trauma and takes initial precedence over all other clinical considerations. There are fundamental differences in the management of casualties arriving at treatment facilities with blunt trauma versus penetrating injury to the face. Patients with isolated blunt trauma to the face and neck, typically undergo standard spinal immobilization measures (in-line traction) during initial airway management. Penetrating maxillofacial and neck injuries often pose significant airway visualization challenges, and the primary priority in such patients, with immediate risk of airway obstruction, is securing a stable airway. Studies performed in civilian and combat settings suggest that patients with normal neurological motor function will not suffer from a mechanically destabilized spinal column that may be compromised by head manipulation during airway control following isolated penetrating trauma to the neck [35].

Initial casualty assessment determines whether the airway is patent and protected, and if breathing is present and adequate. Normal speech may suggest that the airway is patent, at least for the time being, while wheezy breathing usually indicates partial airway obstruction by the tongue and hoarseness or stridor usually suggests a laryngeal cause for airway obstruction. The oral cavity must be thoroughly cleared, visualized and inspected under proper lighting, taking care not to extend or rotate the neck. Blood, loose and fractured teeth, foreign bodies, dirt and mucus are meticulously removed. The midface and mandible are inspected for structural integrity and the anterior neck inspected for penetrating wounds. Crepitus upon palpation of the neck may suggest airway injury or communicating pneumomediastinum. Diminished breath sounds on auscultation may result from atelectasis, pneumothorax, hemothorax or pleural effusion. Wheezing and dyspnea may imply lower airway obstruction, agitation may represent hypoxia at tissue level and cyanosis indicates arterial blood hypoxemia. Suprasternal, supraclavicular or intercostal retractions may be indicative of respiratory muscle fatigue. In the severely ill or unconscious trauma patient, protective airway mechanisms may be impaired or lost, predisposing the patient to aspiration of foreign bodies, loose teeth, bone fragments, secretions, blood and gastric contents. Clearing the airway is the first step in airway management. The chin-lift with head-tilt maneuver opens the airway, but should be avoided in any patient with a potentially unstable cervical spine injury. The jaw thrust without head-tilt maneuver may be performed while maintaining cervical spine alignment and should be utilized in such instances. Between 2–4 % of patients suffering from maxillofacial fractures resulting from blunt trauma, have concomitant cervical spinal fractures, and 10–12 % have cervical ligamentous injuries [33]. Oral or nasopharyngeal airway devices can establish a temporary nonsurgical secure airway in a spontaneously breathing patient, but they do not provide definitive airway protection against aspiration. Nasal airways should not be placed in cases of midface and craniofacial injuries, as they can inadvertently be introduced into the cranial vault in skull base injuries. They are commonly used in semiconscious patients who do not tolerate the oropharyngeal airway, or when the oropharyngeal airway is obstructed (trismus, wiring of teeth etc.). The oropharyngeal airway is designed to thrust the tongue off the posterior pharyngeal wall, thus providing an air channel and suction conduit through the oral cavity. In a conscious patient with an active gag reflex it is contraindicated, as it may stimulate vomiting, laryngospasm and agitation. They are useful in assisting bag-valve-mask ventilation in patients with respiratory failure. All patients with significant blunt trauma to the head or face are at risk for cervical spine injury. Careful consideration for the existence of an unstable spinal injury must be maintained throughout the process of securing the airway [36]. Inadvertent mobilization of the neck of a patient with an unstable cervical spine injury can lead to permanent neurologic injury or death. Accordingly, trauma casualties, particularly with head and neck injuries, are transported with adequate spinal immobilization. An effective approach to airway management with possible cervical spine injuries is rapid sequence intubation with inline spinal immobilization. Pharmacologically paralyzing the patient reduces the risk of patient movement during intubation attempts, improves airway visualization, and facilitates subsequent endotracheal intubation. An alternative airway securing system (surgical airway) should be ready at hand before paralyzing the patient in the event that endotracheal intubation is not possible, but must be used only as last resort, when conservative airway management techniques fail. A definitive airway requires an endotracheal tube (ETT) or tracheostomy tube in the trachea with the cuff inflated, secured in place, and attached to an oxygen-enriched ventilation device. The reasons for securing a definitive airway include: 1) Failure of ventilation or oxygenation; 2) inability to maintain or protect an airway; 3) potential for patient status deterioration; 4) facilitation of patient management during evacuation and transport. The preparation for endotracheal intubation includes: adequate suction, oxygen flow mask, airway management sets (including alternative airway devices such as cricothyroidotomy and tracheostomy), pharmacological agents, a ventilator and monitoring equipment. Ideally, the patient should be preoxygenated with 100 % high-flow oxygen to enable several attempts at intubation without need for intermittent bag-valve-mask ventilation. Cardiac monitoring, blood pressure monitoring and pulse oximetry (as well as end-tidal CO2 monitoring, if available) are mandatory for all patients.

##### Rapid sequence intubation

Given that most patients requiring emergent intubation have not fasted and may have full stomach, bag-valve-mask ventilation may inadvertently lead to gastric distention and passive regurgitation, thus increasing the risk of aspiration. The patient should be preoxygenated first, followed by administration of an induction agent and a rapidly acting neuromuscular blocking agent to induce a state of unconsciousness and paralysis. An assistant should apply cricoid pressure to prevent passive regurgitation of gastric contents, until the ETT has been placed, its position verified and the cuff inflated.

##### Awake endotracheal intubation

Requires liberal topical airway anesthesia and sedation prior to intubation. This permits patent airway reflexes and spontaneous breathing in a patient with a distorted airway caused by displaced mandibular fractures and soft tissue edema.

##### Crash endotracheal intubation

Immediate endotracheal intubation without the use of medications. It is indicated in patients with respiratory arrest, agonal respirations, or deep unresponsiveness. Its advantages are technical ease and rapidity, and the disadvantages are potential increase in intracranial pressure, as well as emesis and aspiration.

##### Confirmation of endotracheal tube placement

As inadvertent intubation of the esophagus may occur during airway management, and may result in patient death. Proper positioning of the ETT within the trachea must be confirmed immediately following intubation. Proper ETT placement detection methods include: clinical assessment, auscultation over the lungs, aspiration of air through the tube, pulse oximetry, end-tidal CO2 level measurement, ultrasonography and chest radiography.

##### Surgical airway management

Despite active bleeding and gaping facial wounds, the fastest and most straightforward technique to establish a definitive airway is often direct laryngoscopy and oral endotracheal intubation. If direct laryngoscopy to achieve endotracheal intubation for immediate airway fails, a cricothyroidotomy is an expedient technique to establish a definitive airway. Alternative surgical airway by tracheotomy requires a formal surgical setting, proper patient positioning, surgical assistance, adequate lighting and suction. In an emergency situation, surgical cricothyroidotomy is preferred to a tracheotomy because it is more rapidly executed, the distance from the skin is shorter (10 mm vs. 20–30 mm) and the risk of violating the vascular thyroid isthmus (resulting in hemorrhage) is lower [37].

##### The difficult airway

The inability to bag-valve-mask ventilate a patient is a contraindication to administering a muscle paralyzing agent (rapid sequence induction). The use of ketamine with atropine may facilitate direct laryngoscopy. A surgical cricothyroidotomy under ketamine anesthesia is another alternative as fiberoptic laryngoscopy. When possible preoxygenation with 100 % highflow oxygen should be performed for several minutes before airway maneuvers.

#### Initial management considerations in maxillofacial trauma

Once a definitive airway is established, active bleeding may be controlled using nasal packs, oropharyngeal packs and a pressure dressing wrapped around the head in cases of profuse bleeding. Epistaxis is often encountered in maxillofacial trauma and is usually managed by primary compression using tamponades [38, 39]. The goal is to slow down blood loss to a level that it can be replaced by blood products, and allow the patient to be fully assessed and stabilized before being transferred to the operating theatre. Patients with suspected ocular injuries should not have compression dressings applied, pending exclusion of globe rupture and penetrating ocular injuries. Mandibular fractures are bandaged in proper maxillo-mandibular occlusal relations for stabilization. Wiring of the teeth together compromises the airway and should usually be avoided. Avulsed teeth and loose bone fragments should be discarded, as should loose dentoalveolar fragments suspended in the airway, to prevent aspiration of loose dentition or bone fragments. Complete avulsion of the ears, nose and lips cannot usually be reattached without readily available microvascular surgery. Partially avulsed structures should be maintained and kept under proper conditions for a later attempt at reconstruction. Parotid gland and duct and facial nerve branches should also be cautiously cleaned, packed open and preserved for later inspection and repair. Facial lacerations should also be cleaned and packed for later closure. Reduction and fixation of craniofacial fractures often represents the best treatment of hemorrhage. Hemorrhage control must be performed aggressively, while judiciously making every effort to prevent further injury of the supraclavicular nervous system (brain and cervical spine), the eyes and other anatomical structures (major blood vessels, cranial nerves, salivary glands etc.) frequently involved in maxillofacial and neck injuries [40, 41]. Severe cases of hemorrhage in which localized pressure does not achieve hemostasis may be remedied with open surgical hemostasis with vascular ligation, bipolar electrocoagulation or using angiographic embolization [42, 43]. Definitive maxillofacial surgery in patients who have undergone temporary fracture stabilization and soft tissue management may be deferred for 7–10 days after the injury. No compromise of functional and esthetic results and no increased risk of postoperative infection and other complications were noted in such patients [44]. Some maxillofacial injuries, however, warrant immediate attention and must be addressed during initial patient care. These injuries may be defined as “maxillofacial medical emergencies” and include unstoppable bleeding from fractures which require immediate reduction and osteosynthesis and intraorbital or intracranial damage to the optic nerve, which necessitates therapeutic action within a few hours [45]. Retrobulbar hematomas, elevated intra-ocular pressure and direct compression of the optic nerve with secondary vision deterioration may necessitate the immediate administration of corticosteroids in high doses over 48 h and/or immediate decompression of the optic nerve [45].

#### Initial assessment and management of neck wounds

Casualties with apparent signs of significant neck injury, including active pulsatile hemorrhage, expanding hematoma, bruit, pulse deficit, subcutaneous emphysema, hoarseness, stridor, respiratory distress or hemiparesis, require urgent surgical consultation and immediate operative management. The first priority in penetrating neck trauma is to assess and secure the airway, keeping in mind the potential for concomitant cervical spine injury. Rigorous spinal precautions should not be maintained at the expense of managing life-threatening airway or vascular injuries. Orotracheal intubation is the initial method of choice for securing the airway under most circumstances. Nasal intubation and fiberoptic intubation techniques are technically more difficult to perform and require special equipment and should therefore be reserved for more difficult cases. Cricothyroidotomy is the preferred method for establishing an immediate airway in cases where rapid endotracheal intubation is not possible or contraindicated. Emergency tracheotomy is the preferred method of establishing a definitive airway in case of suspected tracheal disruption. Attempts at endotracheal intubation could convert a partial tracheal disruption into a complete transection. Casualties who appear to have a minor neck wound may still possess an underlying significant injury. Patients showing signs of stridor, hoarseness, dysphonia, dyspnea, hemoptysis and subcutaneous emphysema should be thoroughly examined for significant injuries to deeper cervical structures. In difficult intubation patients with large anterior penetrating neck wounds, urgent airway can be established by direct intubation of the trachea through the wound.

##### Bleeding

Vascular injury is noted in 20 % of cases of penetrating neck trauma, and exsanguinating hemorrhage is the primary cause of death in such cases [46]. Life threatening facial hemorrhage in maxillofacial surgery has an approximate incidence of 1 % in the trauma patient [47]. Conservative measures such as anterior and posterior nasal packing are recommended as first attempt to control traumatic midface hemorrhage. Temporary reduction of facial fractures is also effective in control of massive facial hemorrhage. Zone II neck injuries with hard signs of vascular injury require immediate surgical exploration. Vascular injuries in zone II mandate a high index of suspicion towards tracheal and esophageal injuries. Suspected proximal (Zone I) carotid injuries require partial sternotomy for proximal control. Injuries to the common or internal carotid arteries may be repaired using lateral arteriography, patch angioplasty, end to end anastomosis or bypass [48]. If the patient is in extremis, the common or internal carotid vessels may be ligated. This approach leads to dismal outcomes, with stroke rates exceeding 20 % and mortality approaching 50 % [49]. An alternative approach to ligation would be the placement of a temporary shunt between the two ends. In case of distal internal carotid (Zone III) injury, ligation is appropriate if the distal end can be also ligated. In case the distal end is within the skull base, a size 3 Fogarty embolectomy catheter can be used to occlude the distal end allowing it to thrombose [50]. Embolization of the bleeding vessel was also recommended as the most reliable technique for the control of hemorrhage [47]. External carotid artery injuries may be repaired using standard techniques or ligated. Transposition of external to internal carotid artery is particularly useful when the internal carotid artery cannot be repaired, as an alternative treatment method to ligation [50].

Hard signs mandating immediate exploration of the neck:

A. Uncontrollable hemorrhage
B. Rapidly expanding hematoma
C. Palpable thrill or audible bruit
D. Focal neurological compromise
E. Absent or decreased pulses in the neck or upper extremities

Bleeding vessels in the neck should never be blindly clamped or probed. Most survivable hemorrhage from neck wounds can be controlled with direct pressure over the wound against the vertebral column, or by packing the wound with gauze. Pharyngeal packing is effective after the airway has been secured. Injuries that do not penetrate the platysma do not cause significant injuries. If violation of the platysma is uncertain, manual spreading of wound edges without probing and visual inspection of the platysma integrity is recommended. Intravenous access should be attained on contralateral side or in the lower torso/extremities. It is useful to triage patients with penetrating neck injuries as either symptomatic or asymptomatic. Symptomatic injuries require immediate surgical exploration, while asymptomatic patients maybe observed pending completion of appropriate studies guided by the location of the wound and availability of resources [50]. Following primary survey and stabilization of other injuries, secondary survey of the entire head and neck region is performed along with appropriate imaging and administration of broad-spectrum antibiotics.

## Conclusions

Severe maxillofacial and neck trauma exposes patients to life threatening complications requiring rapid actions and a strong interplay between surgeons and anesthesiologists. All personnel involved in the care of these patients should take into account DCS and DCR principles.
